# Risk factors for acute kidney injury in patients with acute pancreatitis and construction of nomogram model: a single-center study and external validation

**DOI:** 10.3389/fmed.2025.1626664

**Published:** 2025-09-01

**Authors:** Fan Chen, Kedong Xu, Yimin Han, Jiachun Ding, Jiaqiang Ren, Fang Cao, Yaochun Wang, Weikun Qian, Zheng Wang, Zheng Wu, Zhenhua Ma

**Affiliations:** ^1^Department of Hepatobiliary Surgery, The First Affiliated Hospital of Xi’an Jiaotong University, Xi'an, China; ^2^Pancreatic Disease Center of Xi'an Jiaotong University, Xi'an, China; ^3^Center for Translational Medicine, The First Affiliated Hospital of Xi’an Jiaotong University, Xi'an, China

**Keywords:** acute pancreatitis, acute kidney injury, risk factors, nomogram, MIMIC-IV database

## Abstract

**Objective:**

This study aims to utilize clinical data from patients with acute pancreatitis (AP) recorded in the MIMIC-IV database to analyze the risk factors associated with acute kidney injury (AKI) and to develop a nomogram prediction model.

**Methods:**

This study included clinical data from 754 patients diagnosed with AP sourced from the MIMIC-IV database. They were randomly divided into a training set and an internal validation set. Another 202 patients from the First Affiliated Hospital of Xi’an Jiaotong University were used as an external validation set. Univariate and multivariate logistic regression analyses were conducted to identify the independent influencing factors associated with AKI in these patients. A nomogram model was developed to predict the incidence of AKI, and its performance was evaluated using the area under the receiver operating characteristic curve (AUC), calibration curve, and decision curve analysis (DCA).

**Results:**

Six independent risk factors were identified as predictors of AKI incidence in patients with AP and utilized to construct the nomogram model. The AUC values for the training set, internal validation set, and external validation set were 0.770 (95% CI, 0.719–0.821), 0.755 (95% CI, 0.676–0.834), and 0.628 (95% CI, 0.551–0.706), respectively. Furthermore, the calibration curve indicates that the predicted outcomes align well with the actual observations. Finally, the DCA demonstrates that the nomogram model possesses significant clinical applicability.

**Conclusion:**

The nomogram developed in this study for predicting the incidence of AKI in patients with AP demonstrates strong predictive value and clinical applicability, thereby offering clinicians a more accurate and practical tool for prediction.

## 1 Introduction

Acute pancreatitis (AP) is a prevalent condition characterized by acute abdominal pain within the digestive system. It arises from the abnormal activation of pancreatic enzymes, which can lead to autodigestion of the pancreas and surrounding organs (1). The condition is primarily marked by a localized inflammatory response in the pancreas, potentially resulting in organ damage. In recent years, the incidence of AP has been increasing (2). According to the revised Atlanta classification (RAC), AP is categorized into mild acute pancreatitis (MAP), moderately severe acute pancreatitis (MSAP), and severe acute pancreatitis (SAP) (3). Notably, SAP comprises 5 to 10% of all cases.

Acute kidney injury (AKI) is a common complication associated with AP, with an incidence rate of 10–42% (4). The mortality rate for patients with AP complicated by AKI can be as high as 80% (5). And research indicates that the AKI increases the mortality rate of patients with AP by approximately threefold (6). Patients with AP complicated by AKI have a higher mortality rate, longer hospital stays, and greater hospitalization costs compared to those without AKI (7, 8). Furthermore, among AP patients with concomitant AKI, the survival rates in the surgical intensive care unit and during hospitalization are only 23 and 21% of the rates in AP patients without AKI (9). Therefore, the early and accurate identification of AP complicated by AKI, along with timely intervention measures, is crucial for improving the prognosis of the disease.

Several previous studies have investigated the factors contributing to AKI in patients with AP and have developed predictive models (10–12). However, these studies are characterized by small sample sizes and limited accuracy in their predictive models. Consequently, in clinical practice, the early and accurate diagnosis of AKI in patients with AP continues to pose significant challenges.

This study aimed to identify the risk factors associated with AP concurrent with AKI using a large database. Furthermore, we developed and validated a predictive nomogram model, which is intended to assist clinicians in the early identification of high-risk groups.

## 2 Materials and methods

### 2.1 Data sources

The study data of training set and internal validation set were derived from MIMIC-IV (version 2.2), a large, single-center open critical care database. This database encompasses records of 73,181 patients who were admitted to various intensive care units at Beth Israel Deaconess Medical Center in Boston, Massachusetts, between 2008 and 2019 (13). It contains comprehensive patient records, including demographic indicators, vital sign readings, laboratory results, imaging findings, surgical procedures, medication records, and patient survival status. Additionally, the database includes International Classification of Diseases (ICD-9 and ICD-10) codes, which provide a standardized framework for systematic classification.

The Institutional Review Boards of Beth Israel Deaconess Medical Center and the Massachusetts Institute of Technology approved the utilization of data from the MIMIC-IV database. Informed consent was not necessarily due to the confidential nature of the data. To gain access to the database, we initially completed the mandated online courses and an examination (Record ID: 60630337).

A total of 202 patient records were utilized as the external validation set, sourced from the surgical ICU of the First Affiliated Hospital of Xi’an Jiaotong University. And informed consent was obtained from each patient included in the study. The study protocol conforms to the ethical guidelines of the 1975 Declaration of Helsinki (6th revision, 2008).

### 2.2 Patients and data variables

Data were extracted using Structured Query Language (SQL) programming in PostgreSQL (version 14.0). The SQL script used to extract patient information was obtained from the GitHub repository^1^ (14). Utilizing the International Classification of Diseases (ICD), Ninth Revision (ICD-9, code 577.0), and Tenth Revision (ICD-10, code K85%), we identified patients diagnosed with AP from the MIMIC-IV 2.2 database. Following the identification of eligible patients, we extracted information including demographic data, past medical history, laboratory indicators, interventions, disease severity scores, and survival status. Laboratory parameters were recorded as the first values within the first 24 h after ICU admission, while interventions and disease severity scores were assessed within the same 24-h period.

The assessment of AKI grade is conducted in accordance with the 2012 version of the Kidney Disease Improving Global Outcomes (KDIGO) guidelines (15). The diagnostic criteria were as follows: an increase in serum creatinine (SCr) levels by ≥26.5 μmol/L (0.3 mg/dL) within a 48 h period; an increase in SCr values by ≥50% compared to the baseline value (resulting in a 1.5-fold increase); or a urinary output less than 0.5 mL/kg/h for more than 6 h. Baseline SCr was defined as the lowest value of SCr recorded during prior physical examinations or the SCr measurement obtained 24 h prior to admission.

Inclusion criteria for this study were as follows: (1) patients aged 18 years or older; (2) adherence to the 2012 Atlanta criteria for AP (3); (3) diagnosis of AKI based on the Kidney Disease Improving Global Outcomes (KDIGO) guidelines; and (4) completeness of clinical data. Exclusion criteria included: (1) an intensive care unit (ICU) stay of less than 24 h; (2) serum creatinine measurements taken fewer than two times; (3) patients diagnosed with chronic pancreatitis or pancreatic tumors; (4) patients with pancreatic trauma; (5) pregnant patients; and (6) patients with a history of renal insufficiency. For individuals with multiple ICU admissions, data were collected solely from the first admission.

### 2.3 Development and validation of the nomogram model

The MIMIC database exhibits a significant amount of missing data. In this study, variables with missing values exceeding the 20% threshold were deliberately excluded. Appendix 1 provides a comprehensive overview of the variables and their corresponding proportions of missing values. The trimming method was employed to handle outliers, while multiple imputation techniques were utilized to fill in the missing data (16).

**Figure 1:**
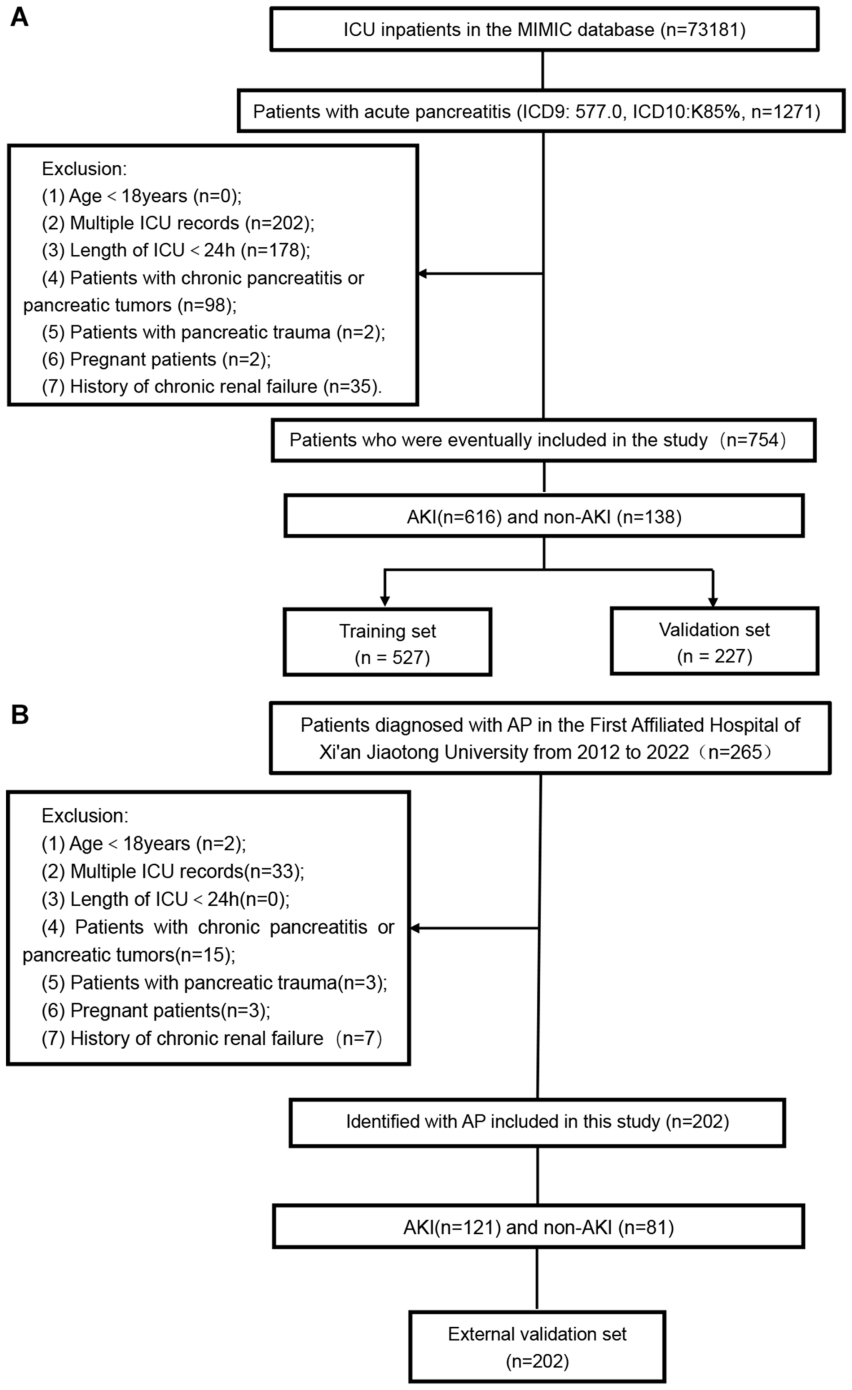
Patients’ inclusion and exclusion flow chart. **(A)** MIMIC database patient screening flow chart; **(B)** The flow chart of patient screening in our hospital.

A total of 754 patients were included in the MIMIC database, which were randomly divided into a training set (*n* = 527) and an internal validation set (*n* = 227) in a 7:3 ratio. Additionally, 202 patients from our institution were included as an external validation set. Subsequently, a nomogram was established based on the training set and underwent both internal and external validation in the validation cohort. The screening process is illustrated in Figure 1.

### 2.4 Statistical analysis

In this study, Excel 2019 was utilized for data organization, while SPSS 22.0 and R 4.1.1 were employed for statistical analysis. Logistic regression analysis was conducted to identify the independent factors influencing AP complicated by AKI. The odds ratio (OR) and corresponding 95% confidence interval (CI) for each variable were calculated. A *p*-value of less than 0.05 was deemed statistically significant.

R software version 4.1.1 was employed for simple random sampling, resulting in the random allocation of patients into training and validation sets. In this study, the ‘rms’, ‘pROC’, and ‘foreign’ software packages were utilized to construct a nomogram, while generating the receiver operating characteristic (ROC) curve to evaluate the discriminative ability of the model. The ‘rms’ package was employed to draw calibration curves to assess the calibration of the model; decision curve analysis (DCA) was conducted using the ‘rmda’ and ‘ggplot2’ package to evaluate its clinical utility. The Bootstrap method, with self-sampling set to B = 1,000, was applied for both internal and external validation in the training and validation sets, respectively. The area under the ROC curve (AUC) was used to evaluate the predictive value of the nomogram, with a minimum value of 0.5 and a maximum value of 1.0; a larger AUC indicates a higher predictive value.

## 3 Results

### 3.1 Patient characteristics

This study included a total of 956 patients who met the inclusion and exclusion criteria. There were three cohorts, including training set (*n* = 527), internal validation set (*n* = 227), and external validation set (*n* = 202). Among the participants, 580 (60.67%) were male, with an average age of 57.29 years. The patients were categorized into two groups based on the development of AKI within 7 days of admission to the ICU: the AKI group and the non-AKI group. The overall incidence of AKI was 77.09% (737/956). Within this cohort, 129 patients (13.49%) were classified as stage 1 AKI, 280 patients (29.29%) as stage 2, and 328 patients (34.31%) as stage 3. The characteristics of the training and validation sets are presented in Table 1. The analysis of the differences between the variables of the included training set and the external validation set is shown in Appendix 2.

**Table 1:** Baseline characteristics of the training and validation sets.

Variables	Total (*n* = 956)	Training set (*n* = 527)	Internal validation set (*n* = 227)	External validation set (*n* = 202)
Age (years)	57.29 ± 17.56	59.35 ± 17.19	59.55 ± 18.23	49.35 ± 15.43
Male, n (%)	580 (60.67)	308 (58.44)	141 (62.11)	131 (64.85)
Hyperlipidemia, n (%)	316 (33.05)	163 (30.93)	59 (25.99)	94 (46.53)
Hypertension, n (%)	424 (44.35)	248 (47.06)	103 (45.37)	73 (36.14)
Diabetes, n (%)	284 (29.71)	168 (31.88)	72 (31.72)	44 (21.78)
Obesity, n (%)	156 (16.32)	65 (12.33)	36 (15.86)	55 (27.23)
Smoking, n (%)	160 (16.74)	70 (13.28)	28 (12.33)	62 (30.69)
Drinking, n (%)	142 (14.85)	63 (11.95)	33 (14.54)	46 (22.77)
Antibiotics, n (%)	842 (88.08)	455 (86.34)	197 (86.78)	190 (94.06)
Vasoactive drugs, n	803 (84.00)	493 (93.55)	216 (95.15)	94 (46.53)
CKD, n (%)	135 (14.12)	87 (16.51)	39 (17.18)	9 (4.46)
SIRS, n (%)	584 (61.09)	399 (75.71)	168 (74.01)	17 (8.42)
Sepsis, n (%)	546 (57.11)	351 (66.60)	164 (72.25)	31 (15.35)
ACS, n (%)	46 (4.81)	18 (3.42)	6 (2.64)	22 (10.89)
Shock, n (%)	276 (28.87)	174 (33.02)	70 (30.84)	32 (15.84)
Ventilation, n (%)	492 (51.46)	270 (51.23)	107 (47.14)	115 (56.93)
RRT, n (%)	187 (19.56)	79 (14.99)	36 (15.86)	72 (35.64)
SOFA score	2.47 ± 2.99	2.00 ± 2.40	2.06 ± 2.75	4.16 ± 3.90
CCI score	3.39 ± 2.78	3.89 ± 2.76	4.11 ± 2.88	1.26 ± 1.31
WBC (x10^9^/L)	13.86 ± 7.90	13.67 ± 7.86	14.75 ± 8.73	13.34 ± 6.92
HCT (%)	34.66 ± 7.97	34.16 ± 7.31	34.01 ± 7.47	36.66 ± 9.71
PLT (x10^9^/L)	208.08 ± 124.97	215.67 ± 131.35	216.09 ± 132.41	179.29 ± 91.03
Blood glucose (mg/dL)	172.31 ± 169.80	160.61 ± 118.75	161.86 ± 126.97	214.59 ± 282.26
Total bilirubin (mg/dL)	2.64 ± 4.63	2.45 ± 4.05	3.22 ± 6.63	2.48 ± 3.01
PT(s)	16.57 ± 9.42	16.91 ± 11.27	16.22 ± 7.34	16.10 ± 5.30
APTT(s)	36.37 ± 17.36	35.82 ± 20.02	34.17 ± 12.73	40.28 ± 13.42
SCr (mg/dL)	1.74 ± 1.83	1.73 ± 1.85	1.73 ± 1.88	1.80 ± 1.74
BUN (mg/dL)	30.62 ± 26.27	30.33 ± 27.55	27.84 ± 23.67	34.51 ± 25.29
Calcium (mg/dL)	7.87 ± 1.27	7.90 ± 1.12	7.91 ± 1.08	7.74 ± 1.76
Sodium (mmol/L)	138.11 ± 6.07	138.35 ± 6.25	137.83 ± 5.27	137.78 ± 6.41
Potassium (mmol/L)	4.19 ± 0.83	4.17 ± 0.87	4.23 ± 0.86	4.18 ± 0.71
Chlorine (mmol/L)	103.45 ± 7.34	104.24 ± 7.87	103.55 ± 6.43	101.25 ± 6.39
Length of ICU (days)	8.42 ± 11.60	7.53 ± 10.85	6.62 ± 7.98	12.74 ± 15.34
Length of hospital	18.70 ± 19.08	18.22 ± 19.05	19.28 ± 19.13	21.61 ± 22.62
AKI stage, n (%)				
0	219 (22.91)	91 (17.27)	47 (20.70)	81 (40.10)
1	129 (13.49)	76 (14.42)	24 (10.57)	29 (14.36)
2	280 (29.29)	172 (32.64)	75 (33.04)	33 (16.34)
3	328 (34.31)	188 (35.67)	81 (35.68)	59 (29.21)

ACS, abdominal compartment syndrome; CKD, chronic kidney disease; RRT, renal replacement therapy; SIRS, systemic inflammatory response syndrome; SOFA, Sequential Organ Failure Assessment; CCI, Charlson Comorbidity Index.

The clinical data of patients in the AKI group were compared with those of patients in the non-AKI group. The results indicated that, in comparison to the non-AKI group, patients in the AKI group were older and experienced longer hospital stays. Furthermore, patients in the AKI group had a greater prevalence of hyperlipidemia, sepsis, systemic inflammatory response syndrome (SIRS), and shock, along with elevated Sequential Organ Failure Assessment (SOFA) and Charlson Comorbidity Index (CCI) scores, as well as increased blood urea nitrogen (BUN) levels. Additionally, patients in the AKI group were more likely to require mechanical ventilation and renal replacement therapy, utilize antibiotic medications, and experience a longer length of stay in the ICU compared to their non-AKI counterparts. The differences between the AKI and non-AKI groups were statistically significant (*p* < 0.05; Table 2).

**Table 2:** A comparison of the baseline characteristics between the AKI and non-AKI groups.

Variables	Training set (*n* = 527)	Non-AKI group (*n* = 91)	AKI group (*n* = 436)	Statistics	*p*
Age (years)	59.35 ± 17.19	55.21 ± 17.43	60.22 ± 17.03	*t* = −2.54	0.011
Male, n (%)	308 (58.44)	51 (56.04)	257 (58.94)	χ^2^ = 0.26	0.61
Hyperlipidemia, n (%)	163 (30.93)	20 (21.98)	143 (32.80)	χ^2^ = 4.13	0.042
Hypertension, n (%)	248 (47.06)	45 (49.45)	203 (46.56)	χ^2^ = 0.25	0.615
Diabetes, n (%)	168 (31.88)	25 (27.47)	143 (32.80)	χ^2^ = 0.98	0.321
Obesity, n (%)	65 (12.33)	8 (8.79)	57 (13.07)	χ^2^ = 1.28	0.258
Smoking, n (%)	70 (13.28)	20 (21.98)	50 (11.47)	χ^2^ = 7.22	0.007
Drinking, n (%)	63 (11.95)	14 (15.38)	49 (11.24)	χ^2^ = 1.23	0.267
Antibiotics, n (%)	455 (86.34)	68 (74.73)	387 (88.76)	χ^2^ = 12.57	<0.001
Vasoactive drugs	493 (93.55)	83 (91.21)	410 (94.04)	χ^2^ = 1.00	0.318
CKD, n (%)	87 (16.51)	6 (6.59)	81 (18.58)	χ^2^ = 7.85	0.005
SIRS, n (%)	399 (75.71)	59 (64.84)	340 (77.98)	χ^2^ = 7.08	0.008
Sepsis, n (%)	351 (66.60)	43 (47.25)	308 (70.64)	χ^2^ = 18.52	<0.001
ACS, n (%)	18 (3.42)	0 (0.00)	18 (4.13)	χ^2^ = 2.74	0.098
Shock, n (%)	174 (33.02)	9 (9.89)	165 (37.84)	χ^2^ = 26.60	<0.001
Ventilation, n (%)	270 (51.23)	27 (29.67)	243 (55.73)	χ^2^ = 20.47	<0.001
RRT, n (%)	79 (14.99)	0 (0.00)	79 (18.12)	χ^2^ = 19.40	<0.001
SOFA score	2.00 ± 2.40	1.25 ± 1.90	2.15 ± 2.46	*t* = −3.88	<0.001
CCI score	3.89 ± 2.76	2.96 ± 2.52	4.09 ± 2.77	*t* = −3.60	<0.001
WBC (x10^9^/L)	13.67 ± 7.86	12.67 ± 5.82	13.88 ± 8.21	*t* = −1.66	0.098
HCT (%)	34.16 ± 7.31	33.91 ± 5.14	34.22 ± 7.70	*t* = −0.48	0.632
PLT (x10^9^/L)	215.67 ± 131.35	212.59 ± 130.81	216.31 ± 131.60	*t* = −0.25	0.806
Blood glucose (mg/dL)	160.61 ± 118.75	150.24 ± 86.40	162.77 ± 124.42	*t* = −0.92	0.36
Total bilirubin (mg/dL)	2.45 ± 4.05	1.86 ± 2.05	2.57 ± 4.34	*t* = −1.53	0.126
PT(s)	16.91 ± 11.27	13.98 ± 5.83	17.52 ± 12.01	*t* = −2.74	0.006
APTT(s)	35.82 ± 20.02	32.49 ± 10.04	36.52 ± 21.47	*t* = −1.75	0.081
SCr (mg/dL)	1.73 ± 1.85	1.36 ± 1.98	1.81 ± 1.82	*t* = −2.10	0.036
BUN (mg/dL)	30.33 ± 27.55	22.45 ± 26.42	31.98 ± 27.52	*t* = −3.02	0.003
Calcium (mg/dL)	7.90 ± 1.12	7.87 ± 0.90	7.91 ± 1.16	*t* = −0.26	0.798
Sodium (mmol/L)	138.35 ± 6.25	138.24 ± 6.32	138.37 ± 6.24	*t* = −0.18	0.857
Potassium (mmol/L)	4.17 ± 0.87	3.98 ± 0.79	4.21 ± 0.88	*t* = −2.33	0.02
Chlorine (mmol/L)	104.24 ± 7.87	104.70 ± 8.10	104.15 ± 7.83	*t* = 0.61	0.542
Length of ICU (days)	7.53 ± 10.85	2.46 ± 1.35	8.59 ± 11.64	*t* = −10.66	<0.001
Length of hospital	18.22 ± 19.05	10.08 ± 11.40	19.92 ± 19.88	*t* = −6.45	<0.001
AKI stage, n (%)				χ^2^ = 527	<0.001
0	91 (17.27)	91 (100.00)	0 (0.00)		
1	76 (14.42)	0 (0.00)	76 (17.43)		
2	172 (32.64)	0 (0.00)	172 (39.45)		
3	188 (35.67)	0 (0.00)	188 (43.12)		

ACS, abdominal compartment syndrome; AKI, acute kidney injury; CKD, chronic kidney disease; RRT, renal replacement therapy; SIRS, systemic inflammatory response syndrome; SOFA, Sequential Organ Failure Assessment; CCI, Charlson Comorbidity Index.

### 3.2 Analysis of risk factors of AP complicated by AKI

Among the 527 patients in the training set, 436 were complicated by AKI. For the continuous variables in this study, we performed linear analyses and plotted restricted cubic spline (RCS) curves, the results of which are shown in Appendix 3. For the continuous variable PT in this study, the Box-Tidwell test was performed, and its *p* value was 0.7319, indicating that the relationship between this variable and logit (P) was linear. At the same time, we plot the scatter plot for observation, as shown in Appendix 4. At the same time, Pearson correlation analysis was used to evaluate the collinearity between variables, and the results are shown in Appendix 5.

Univariate analysis was conducted on the patients in the training set, revealing that age, hyperlipidemia, smoking, antibiotic use, coexisting chronic kidney disease (CKD), systemic inflammatory response syndrome (SIRS), sepsis, shock, mechanical ventilation, the Sequential Organ Failure Assessment (SOFA) score, the Charlson Comorbidity Index (CCI) score, prothrombin time (PT), serum creatinine (SCr), blood urea nitrogen (BUN), and high serum potassium are all significant influencing factors for AP patients with AKI (*p* < 0.05). Refer to Table 3. To exclude multicollinearity in logistic regression, we performed variance inflation factor (VIF) analysis, and the results are shown in Appendix 6.

**Table 3:** Results of the univariate analysis showing the risk factors of AP complicated by AKI.

Variables	Univariate analysis				
	β	S. E	Z	*p*	OR (95%CI)
Age (years)	0.02	0.01	2.51	0.012	1.02 (1.01–1.03)
Gender (Male)	0.12	0.23	0.51	0.61	1.13 (0.71–1.78)
Hyperlipidemia (Yes)	0.55	0.27	2.01	0.044	1.73 (1.01–2.96)
Hypertension (Yes)	−0.12	0.23	−0.5	0.615	0.89 (0.57–1.40)
Diabetes (Yes)	0.25	0.26	0.99	0.322	1.29 (0.78–2.13)
Obesity (Yes)	0.44	0.4	1.12	0.262	1.56 (0.72–3.39)
Smoking (No)	−0.78	0.29	−2.64	0.008	0.46 (0.26–0.82)
Drinking alcohol (Yes)	−0.36	0.33	−1.1	0.27	0.70 (0.37–1.32)
Antibiotics (Yes)	0.98	0.28	3.45	<0.001	2.67 (1.53–4.67)
Vasoactive drugs (Yes)	0.42	0.42	0.99	0.321	1.52 (0.66–3.47)
CKD (Yes)	1.17	0.44	2.67	0.008	3.23 (1.36–7.66)
SIRS (Yes)	0.65	0.25	2.63	0.009	1.92 (1.18–3.12)
Sepsis (Yes)	0.99	0.23	4.21	<0.001	2.69 (1.70–4.26)
ACS (Yes)	15.04	565.58	0.03	0.979	3407353.66 (0.00–Inf)
Shock (Yes)	1.71	0.36	4.7	<0.001	5.55 (2.71–11.34)
Ventilation (Yes)	1.09	0.25	4.39	<0.001	2.98 (1.83–4.86)
RRT (Yes)	17.2	733.85	0.02	0.981	29479104.16 (0.00–Inf)
SOFA score	0.2	0.06	3.18	0.001	1.22 (1.08–1.38)
CCI score	0.17	0.05	3.52	<0.001	1.19 (1.08–1.30)
WBC (x10^9^/L)	0.02	0.02	1.33	0.183	1.02 (0.99–1.05)
HCT (%)	0.01	0.02	0.37	0.71	1.01 (0.98–1.04)
PLT (x10^9^/L)	0	0	0.25	0.806	1.00 (1.00–1.00)
Blood glucose (mg/dL)	0	0	0.91	0.361	1.00 (1.00–1.00)
Total bilirubin (mg/dL)	0.07	0.04	1.51	0.132	1.07 (0.98–1.17)
PT(s)	0.1	0.03	3.12	0.002	1.11 (1.04–1.18)
APTT(s)	0.02	0.01	1.68	0.093	1.02 (1.00–1.03)
SCr (mg/dL)	0.19	0.09	2.05	0.041	1.21 (1.01–1.45)
BUN (mg/dL)	0.02	0.01	2.93	0.003	1.02 (1.01–1.03)
Serum Calcium (mg/dL)	0.03	0.1	0.26	0.798	1.03 (0.84–1.26)
Serum Sodium (mmol/L)	0	0.02	0.18	0.857	1.00 (0.97–1.04)
Serum Potassium (mmol/L)	0.37	0.16	2.32	0.02	1.45 (1.06–1.99)
Serum Chlorine (mmol/L)	−0.01	0.01	−0.61	0.541	0.99 (0.96–1.02)

ACS, abdominal compartment syndrome; CKD, chronic kidney disease; RRT, renal replacement therapy; SIRS, systemic inflammatory response syndrome; SOFA, Sequential Organ Failure Assessment; CCI, Charlson Comorbidity Index.

**Figure 2:**
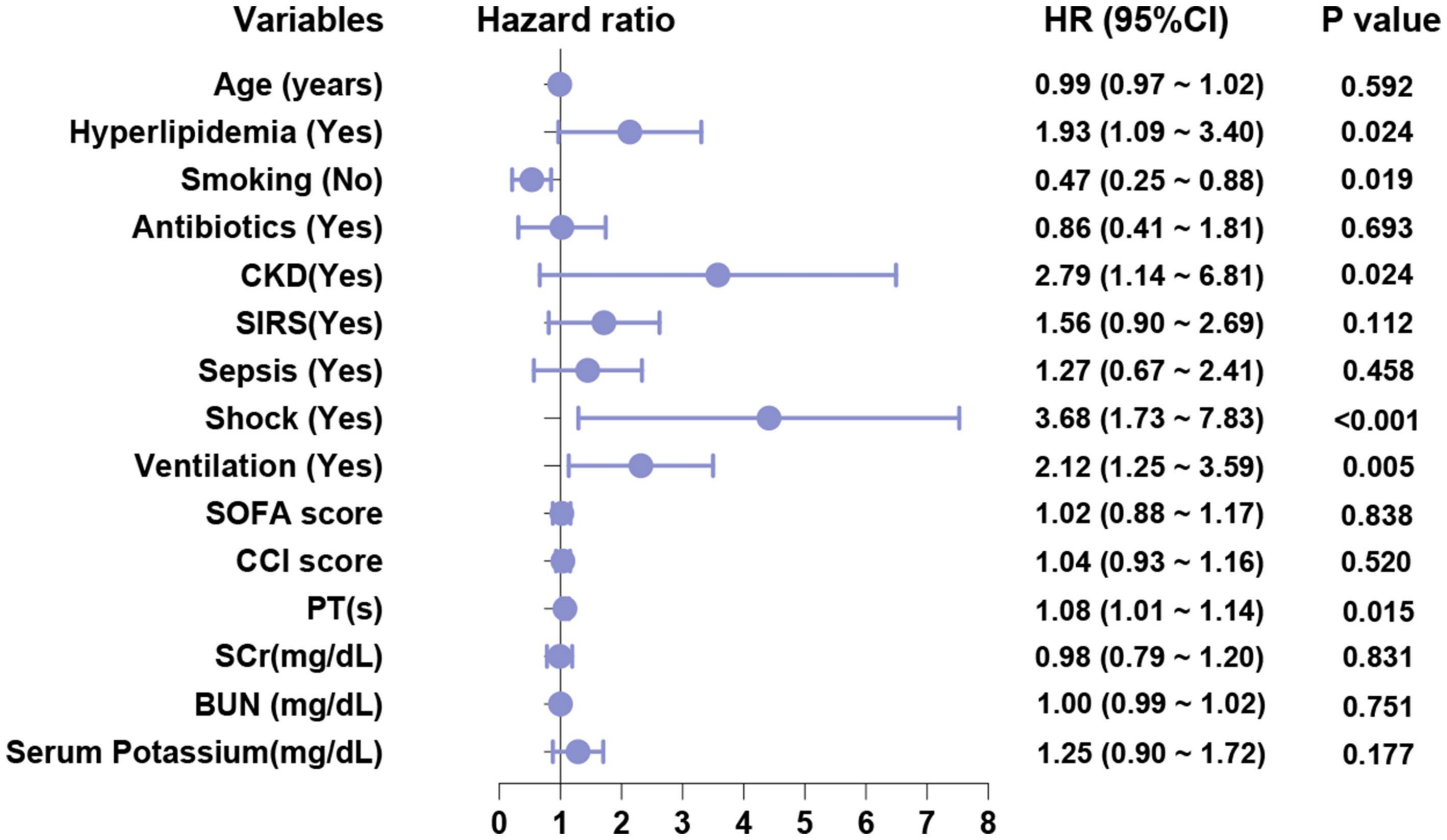
Multivariate regression analysis of forest plots.

Based on the results of the univariate analysis, a multivariate logistic regression analysis was subsequently conducted. The factors included in this analysis were age, hyperlipidemia, smoking, antibiotic use, CKD, SIRS, sepsis, shock, mechanical ventilation, SOFA score, CCI score, PT, SCr, BUN and serum potassium. The results are presented in Table 4 and Figure 2. Among these factors, hyperlipidemia (OR = 1.93, 95% CI = 1.09–3.40, *p* = 0.024), smoking (OR = 0.47, 95% CI = 0.25–0.88, *p* = 0.019), CKD (OR = 2.79, 95% CI = 1.14–6.81, *p* = 0.024), shock (OR = 3.68, 95% CI = 1.73–7.83, *p* < 0.001), mechanical ventilation (OR = 2.12, 95% CI = 1.25–3.59, *p* = 0.005), and PT (OR = 1.08, 95% CI = 1.01–1.14, *p* = 0.015) were identified as significant factors influencing AKI in patients with AP (*p* < 0.05). To exclude potential interactions between variables, we performed a simultaneous subgroup analysis, as shown in Appendix 7, suggesting that hyperlipidemia, smoking, CKD, shock, mechanical ventilation, and PT are independent risk factors affecting the occurrence of AKI in AP patients.

**Table 4:** Results of the multivariate analysis showing the risk factors of AP complicated by AKI.

Variables	Multivariate analysis				
	β	S. E	Z	*p*	OR (95%CI)
Age (years)	−0.01	0.01	−0.54	0.592	0.99 (0.97–1.02)
Hyperlipidemia (Yes)	0.66	0.29	2.26	0.024	1.93 (1.09–3.40)
Smoking (No)	−0.76	0.32	−2.34	0.019	0.47 (0.25–0.88)
Antibiotics (Yes)	−0.15	0.38	−0.39	0.693	0.86 (0.41–1.81)
CKD (Yes)	1.03	0.46	2.26	0.024	2.79 (1.14–6.81)
SIRS (Yes)	0.44	0.28	1.59	0.112	1.56 (0.90–2.69)
Sepsis (Yes)	0.24	0.33	0.74	0.458	1.27 (0.67–2.41)
Shock (Yes)	1.3	0.39	3.38	<0.001	3.68 (1.73–7.83)
Ventilation (Yes)	0.75	0.27	2.78	0.005	2.12 (1.25–3.59)
SOFA score	0.01	0.07	0.2	0.838	1.02 (0.88–1.17)
CCI score	0.04	0.06	0.64	0.52	1.04 (0.93–1.16)
PT(s)	0.07	0.03	2.42	0.015	1.08 (1.01–1.14)
SCr (mg/dL)	−0.02	0.11	−0.21	0.831	0.98 (0.79–1.20)
BUN (mg/dL)	0	0.01	0.32	0.751	1.00 (0.99–1.02)
Serum Potassium (mmol/L)	0.22	0.16	1.35	0.177	1.25 (0.90–1.72)

CKD, chronic kidney disease; SIRS, systemic inflammatory response syndrome; SOFA, Sequential Organ Failure Assessment; CCI, Charlson Comorbidity Index.

As shown in Table 4, the OR values for hyperlipidemia, CKD, shock, mechanical ventilation, and PT are all greater than 1. This indicates that these factors are associated with an increased risk of AKI in patients with AP. Consequently, patients presenting with hyperlipidemia, CKD, shock, the need for mechanical ventilation, and elevated PT values exhibit a higher OR and, therefore, an elevated risk of developing AKI.

### 3.3 Nomogram of AKI in patients with AP

**Figure 3:**
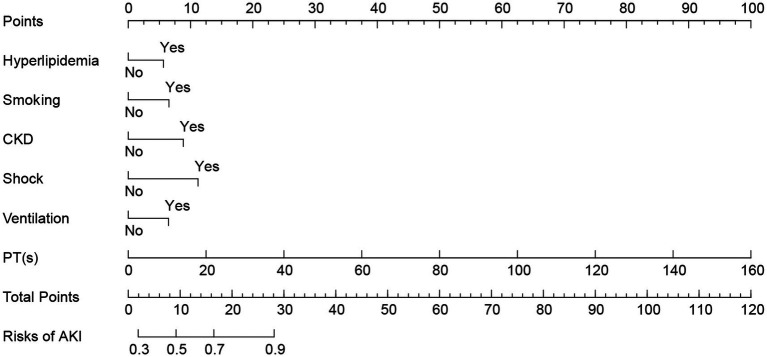
Nomogram of AKI in patients with AP. AP, acute pancreatitis; AKI, acute kidney injury.

Based on the results of a multivariate logistic regression analysis, statistically significant independent predictive factors were integrated to construct a nomogram model. The predictive factors included hyperlipidemia, smoking, CKD, shock, mechanical ventilation, and PT. The prediction results for patients with concurrent AKI are presented in Figure 3.

The line segment associated with each variable in the figure is marked with a scale that indicates the range of values the variable can assume. The length of each line segment reflects the contribution of the factor to the final event. Based on the scores (Points) corresponding to each variable at various values, the individual scores for all variables are summed to yield the total score (Total Points). The incidence rate of AKI can then be determined by projecting this total score downward.

### 3.4 Verification of the nomogram

**Figure 4:**
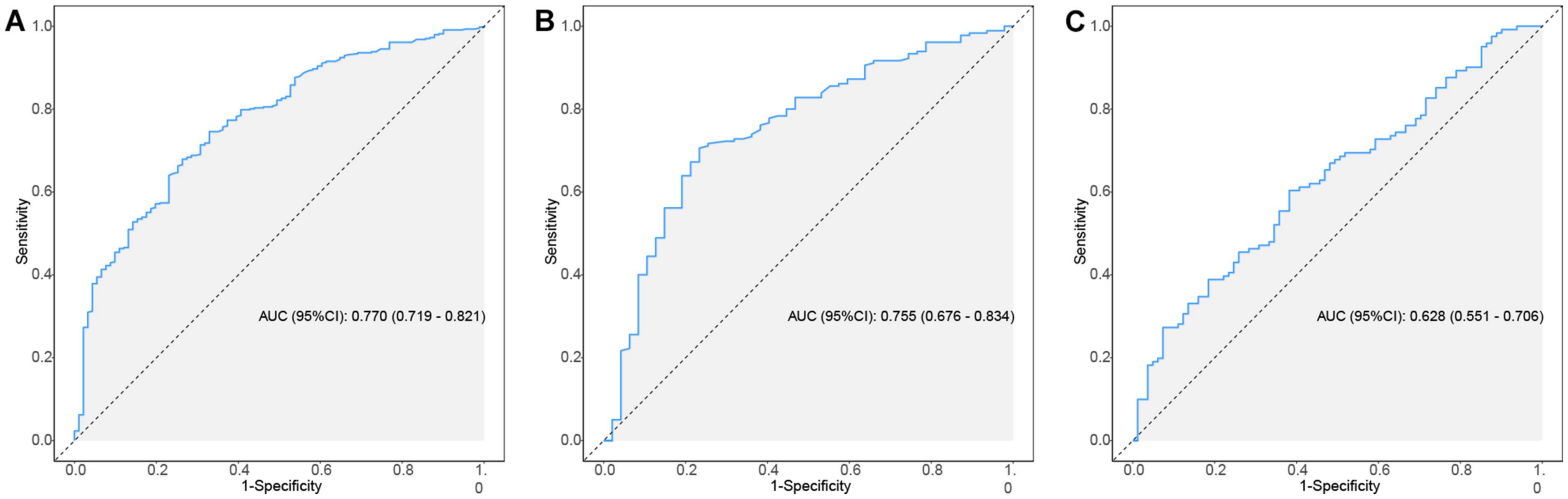
The ROC curves of nomogram predicting AKI in patients of AP. **(A–C)** ROC curves of the nomogram for predicting the likelihood of developing AKI in AP patients in the training set, internal validation set and external validation set. ROC, receiver operating characteristic; AP, acute pancreatitis; AKI, acute kidney injury.

This study employed the ROC curve to assess the discriminative ability of the model. Figures 4A–C illustrate the ROC curves and the AUC values predicted by the nomogram model for the incidence of AKI in patients with AP. In the training and validation sets, the relevant metrics of the ROC curve are shown in Table 5. In the training set, the model achieved an AUC of 0.770 (95% CI, 0.719–0.821), whereas in the internal validation set, the AUC was 0.755 (95% CI, 0.676–0.834). And in the external validation set, the AUC value was 0.628 (95% CI, 0.551–0.706). The nomogram model developed in this study demonstrated good predictive value in both the training and validation sets.

**Table 5:** Information of ROC curves in Figure 4.

Data	AUC (95%CI)	Accuracy (95%CI)	Sensitivity (95%CI)	Specificity (95%CI)	PPV (95%CI)	NPV (95%CI)
Training set	0.770 (0.719–0.821)	0.731 (0.690–0.768)	0.670 (0.574–0.767)	0.743 (0.702–0.784)	0.353 (0.281–0.424)	0.915 (0.886–0.944)
Internal validation set	0.755 (0.676–0.834)	0.722 (0.659–0.780)	0.745 (0.620–0.869)	0.717 (0.651–0.782)	0.407 (0.303–0.511)	0.915 (0.869–0.961)
External validation set	0.628 (0.551–0.706)	0.525 (0.453–0.595)	0.889 (0.820–0.957)	0.281 (0.201–0.361)	0.453 (0.375–0.530)	0.791 (0.669–0.912)

ROC, receiver operating characteristic; PPV, positive predictive value; NPV, negative predictive value.

**Figure 5:**
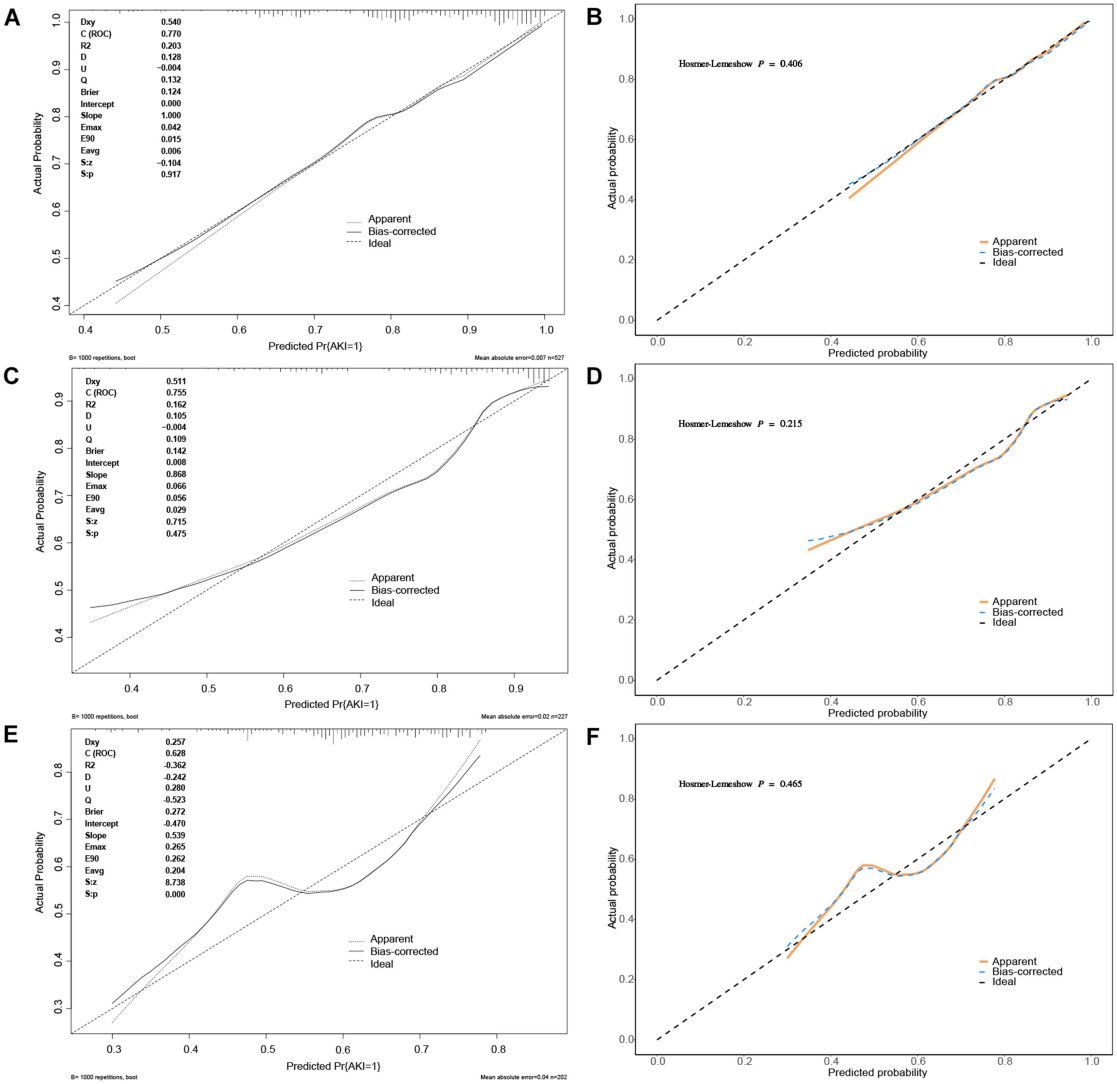
The calibration curves of nomogram predicting AKI in patients of AP. **(A,B)** The calibration curves of nomogram predicting AKI in patients of AP in the training set; **(C,D)** The calibration curves of nomogram predicting AKI in patients of AP in the internal validation set; **(E,F)** The calibration curves of nomogram predicting AKI in patients of AP in the external validation set.

The calibration curve of the nomogram model for predicting the incidence of AKI in patients is presented in Figure 5. As shown in the figure, the Brier scores of the model calibration curves are 0.124, 0.142, and 0.272 in the training set, internal and external validation sets, respectively. The calibration curves for the training set and the validation sets closely align with the ideal 45° dotted line, indicating a strong consistency between the predicted values and the actual observed values.

**Figure 6:**
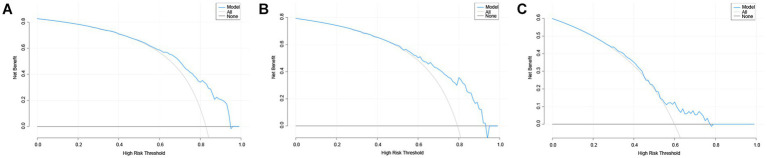
The DCA curves of nomogram predicting AKI in patients of AP. **(A–C)** The DCA curves of nomogram predicting AKI in patients of AP in the training set, internal validation set and external validation set. DCA, decision analysis curve; AP, acute pancreatitis; AKI, acute kidney injury.

The DCA curve of the Nomogram model for predicting the overall survival rate of patients with AP complicated by AKI is presented in Figure 6. The figure illustrates that when the threshold probability for the incidence of AKI in patients ranges from 0.55 to 0.95, the net benefit associated with the application of the nomogram is significantly higher than that of both the “none intervention” and “all intervention” strategies. The clinical scenario corresponding to the threshold probability range of 0.55–0.95 is that when clinicians believe that the probability of AKI in patients exceeds 55%, preventive interventions based on model predictions (such as enhanced surveillance, early kidney protection measures) will bring net benefits. This finding suggests that the nomogram demonstrates strong clinical applicability in predicting the incidence of AKI in patients with AP.

## 4 Discussion

AP is primarily caused by common factors such as biliary tract disease, hyperlipidemia, and excessive alcohol consumption. This condition activates pancreatic enzymes, leading to an acute inflammatory response in pancreatic tissue. As the disease progresses, pancreatic tissue gradually becomes necrotic, with the extent of necrosis progressively increasing. In severe cases, infection of the necrotic pancreatic tissue may occur, often accompanied by failure of other organs. AKI is a frequent complication of AP and is typically associated with a poor prognosis for patients. Clinical practice has demonstrated that AKI is a significant contributor to mortality in patients with AP (9). Currently, apart from renal replacement therapy, there are no effective pharmacological interventions available to mitigate renal damage in patients with acute pancreatitis complicated by AKI (17). Therefore, implementing proactive measures to early identify AP complicated by AKI and to execute effective interventions is critically important for improving patient outcomes.

In our study, both univariate and multivariate logistic regression analyses were employed to thoroughly investigate the risk factors for AKI in patients with AP. The findings indicate that combined hyperlipidemia, CKD, shock, smoking, mechanical ventilation, and PT are independent risk factors for AKI in this patient population, aligning with the results of previous studies.

Patients with hyperlipidemia-related pancreatitis exhibit a higher incidence of AKI, with hypertriglyceridemia identified as an independent risk factor during the early stages of AP. Relevant studies indicate that patients with triglyceride levels exceeding 200 mg/dL have an AKI incidence as high as 87%, in contrast to only 35% among patients with normal triglyceride levels (18). This association may be linked to the pathophysiological mechanisms underlying AP. During an acute pancreatitis episode, the inflammatory response within pancreatic tissue can trigger SIRS, subsequently impairing kidney function. Hypertriglyceridemia may exacerbate this inflammatory response, thereby heightening the risk of AKI (19). Consequently, clinical management is crucial for patients with hypertriglyceridemia-related AP. Early identification and intervention for elevated triglyceride levels can mitigate the incidence of AKI.

CKD is recognized as an independent risk factor for AKI during the early stages of AP. Epidemiological studies have demonstrated that patients with CKD face a significantly heightened risk of developing AKI. Numerous studies have indicated that CKD is prevalent among patients experiencing AKI events, thereby suggesting a strong association between the two conditions. Patients with CKD may exhibit increased sensitivity to AKI due to impaired renal tubular function, a sensitivity that is particularly pronounced in acute illnesses such as AP (20, 21). Furthermore, individuals with CKD often present with renal tubular dysfunction and a reduced glomerular filtration rate (GFR), which further predisposes them to kidney injury during acute episodes. Additionally, increased proteinuria is a notable characteristic of CKD patients and is significantly correlated with the incidence of AKI (22).

Shock is recognized as an independent risk factor for AP complicated by AKI. Research indicates that, in patients with SAP, the occurrence of shock significantly heightens the risk of developing AKI. Various studies have examined multiple factors, including fluid imbalance, inflammatory response, and organ dysfunction, underscoring the critical role of shock in the pathogenesis of AKI (23). In addition to being an independent risk factor, shock interacts with other variables, such as age and underlying health conditions, further exacerbating the risk of AKI (24, 25). The systemic inflammatory response associated with AP may intensify renal damage through the release of cytokines and the activation of immune cells (24). Moreover, the presence of shock may lead to elevated levels of specific biomarkers, such as cytokines, which are strongly correlated with renal dysfunction (26).

In patients with AP, smoking is significantly associated with the occurrence of AKI. Ishigami et al. found that the incidence of AKI in current smokers was notably higher than that in non-smokers. Specifically, data indicate that the risk of AKI in current smokers is twice that of non-smokers, underscoring the critical role of smoking in the development of AKI (27). Furthermore, another study reported that both smokers and former smokers exhibited a significantly higher incidence of AKI during hospitalization for AP compared to never smokers, reinforcing the notion of smoking as a risk factor for AKI (28, 29).

Mechanical ventilation can serve as a predictive factor for AKI in patients with AP, aligning with findings from previous studies (9). Research indicates that acute respiratory failure resulting from AP necessitates mechanical ventilation for patients admitted to the ICU. However, the use of mechanical ventilation may lead to acute lung injury, which can exacerbate hypoxia, induce vasoconstriction, decrease renal perfusion, and diminish the glomerular filtration rate. Furthermore, coagulation dysfunction emerges as an independent risk factor for AKI, with prolonged PT closely associated with the onset of AKI, thus serving as a significant prognostic indicator (24, 30).

Wu et al. found that age is an independent risk factor for the occurrence of AKI in patients with AP. This is due to the correlation between increasing age and declining renal function. As age increases, the incidence of AKI related to the deterioration of physiological functions is higher in patients with AP (31). Furthermore, age is a known predictive factor for the severity and mortality of AP (32). However, the results of the univariate analysis in this study indicate that age is a risk factor influencing the incidence of AKI in patients, while the multifactorial results show that age is not an independent risk factor. This may be due to the collinearity between age and included variables such as CKD and shock. Although age is not an independent predictive factor, caution should still be exercised with elderly patients, as they are more likely to have a synergistic effect from multiple risk factors.

The pathogenesis of AP-related AKI has not yet been fully elucidated. Current studies suggest that it is primarily associated with factors such as insufficient effective circulating blood volume, abnormal hemodynamics, microcirculation disorders, and inflammatory mediators (26, 33). Research has demonstrated that serum procalcitonin can predict the development of AKI in patients with AP and can also be utilized for the dynamic evaluation of AKI prognosis. Its predictive value surpasses that of C-reactive protein (CRP), interleukin-6 (IL-6), and serum amyloid A. Additionally, the ratio of neutrophils to lymphocytes is closely linked to the severity of AP and the impairment of kidney function throughout the disease course, exhibiting high diagnostic efficiency for SAP-related AKI (34). Furthermore, CT-based imaging evaluation holds significant value in diagnosing SAP-related AKI. One study revealed that among various CT indices, the Extra pancreatic Inflammation on CT score (EPIC) demonstrates a strong correlation with the Acute Physiology and Chronic Health Evaluation II score and the Ranson score, thereby providing a better prediction of SAP-related AKI (35).

This study developed a prediction model to forecast the incidence of AKI in patients with AP, utilizing multivariate logistic regression analysis. The nomogram model constructed in this study quantitatively predicts the risk of AKI in patients with AP, holding significant clinical value. By identifying high-risk patients through individualized scoring, it guides enhanced monitoring and preventive interventions. It assists clinicians in formulating differentiated treatment plans, optimizing the allocation of medical resources. Furthermore, it provides an intuitive visualization tool, facilitating the explanation of risks and achieving consensus in diagnosis and treatment. This model is expected to be translated into preventive clinical practice, improving patient outcomes, and can potentially achieve real-time risk assessment through integration with electronic medical record systems in the future.

This study has several limitations. First, as a retrospective cohort analysis, this study indeed has inherent limitations of selection bias and measurement bias. We particularly note that due to the lack of randomization design, the established predictive model can only reflect statistical associations between variables and cannot infer causal relationships. This limitation is consistent with other similar predictive model studies (36). Additionally, due to the missing variables within the MIMIC database itself, some risk factors cannot be included in the study. Therefore, the predictive model developed needs to be validated through prospective studies. Second, the limited number of patients in the database may introduce bias in the results, as data from the single-center MIMIC database could lead to selection bias or other limitations affecting the generalizability of the study findings. Lastly, although the established predictive model demonstrates good discrimination and validation, the AUC is not particularly high. Furthermore, this study lacks multi-center external validation, which may fail to fully capture the heterogeneity of AP patients in other settings. Most importantly, the MIMIC database lacks records for APACHE scores, and the application value of these scores in predicting the incidence of AKI in patients cannot be further verified, so more studies are needed to explore them. MIMIC-IV did not routinely record the core indicators of the Atlanta standard, such as the extent of pancreatic necrosis, so this study did not conduct a further stratified analysis of the severity of AP patients. Failure to perform precise stratification may lead to the model’s prediction bias for patients with severe AP.

In future studies, we will conduct prospective validation studies in different AP patient populations, evaluate the cost-effectiveness of nomograms, and explore the underlying mechanisms by which identified relevant risk factors lead to AKI.

## 5 Conclusion

The nomogram developed in this study for predicting the incidence of AKI in patients with AP demonstrates strong predictive value and clinical applicability, thereby offering clinicians a more accurate and practical tool for prediction.

## Data Availability

The original contributions presented in the study are included in the article/Supplementary material, further inquiries can be directed to the corresponding authors.

## Footnotes

^1^ https://github.com/MIT-LCP/mimic-code/tree/main/mimic-iv
