# Supplementary material for: Risk factors for acute kidney injury in patients with acute pancreatitis and construction of nomogram model: a single-center study and external validation
Source: Front Med (Lausanne). 2025 Sep 1;12:1626664. doi: 10.3389/fmed.2025.1626664 (PMC12434119; doi:10.3389/fmed.2025.1626664)
Supplement: Supplementary file 1 [file Data_Sheet_1.docx]

**Appendix 1.** The proportion of missing values of variables

| Variables | Missing proportions | Variables | Missing proportions |
| --- | --- | --- | --- |
| Patient code | | | |
| subject_id | 0% | hadm_id | 0% |
| stay_id | 0% | icd_version | 0% |
| icd_code | 0% | seq_num | 0% |
| Demographic information and hospitalization information | | | |
| Age | 0% | Height | 40.56% |
| Sex | 0% | Weight | 2.66% |
| Admission time | 0% | Time of discharge | 0% |
| Length of hospital stay | 0% | Length of ICU hospital stay | 0% |
| Survive days from ICU intime | 0% | Survive days from admit time | 0% |
| Comorbid disease | | | |
| Hyperlipidemia | 0% | Hypertension | 0% |
| Diabetes | 0% | Obesity | 0% |
| History of smoking | 0% | History of drinking alcohol | 0% |
| Malignant tumor | 0% | Chronic pancreatitis | 0% |
| Pregnant patients | 0% | SIRS | 0% |
| Chronic kidney disease | 0% | Sepsis | 0% |
| Shock | 0% | End stage renal disease | 0% |
| Interventions (treatment or medical operation performed in the first 24 h after admission to the ICU) | | | |
| Antibiotics | 0% | Mechanical ventilation | 0% |
| Vasoactive drugs | 0% | Renal replacement therapy | 0% |
| Disease severity score (assessed in the first 24 h after admission to the ICU) | | | |
| APACHE II score | 63.37% | SOFA score | 0% |
| CCI score | 0% |  |  |
| Laboratory indicators (first value within the first 24 h of admission to the ICU) | | | |
| White blood cell | 0% | Hematocrit | 0% |
| Platelet | 0% | Neutrophils count | 44.36% |
| Lymphocyte count | 44.23% | Serum lipase | 41.32% |
| Serum amylase | 67.30% | Total bilirubin | 5.31% |
| Direct bilirubin | 84.30% | Indirect bilirubin | 84.30% |
| Total protein | 97.56% | Albumin | 41.06% |
| Blood glucose | 0% | Prothrombin time | 0% |
| Activates partial thromboplastin time | 0% | Triglycerides | 6.70% |
| Total cholesterol | 71.23% | High-density lipoprotein | 65.30% |
| Low-density lipoprotein | 65.30% | Blood urea nitrogen | 0% |
| Serum creatinine | 0% | Serum calcium | 0% |
| Serum sodium | 0% | Serum chlorine | 0% |
| Serum potassium | 0% |  |  |
| Outcome (occurred within 7 days of admission to the ICU) | | | |
| Acute kidney injury (AKI) | 0% | AKI stage | 0% |

**Appendix 2.** Data heterogeneity analysis of training set and external validation set

| Variables | Training set | External  validation set | P value | SMD |
| --- | --- | --- | --- | --- |
|  | (n = 527) | (n = 202) |  |  |
| Age (years) | 59.35 ± 17.19 | 49.35 ± 15.43 | <.001 | -0.648 |
| Male, n (%) | 308 (58.44) | 131 (64.85) | 0.114 | 0.134 |
| Hyperlipidemia, n (%) | 163 (30.93) | 94 (46.53) | <.001 | **0.313** |
| Hypertension, n (%) | 248 (47.06) | 73 (36.14) | 0.008 | -0.227 |
| Diabetes, n (%) | 168 (31.88) | 44 (21.78) | 0.007 | -0.245 |
| Obesity, n (%) | 65 (12.33) | 55 (27.23) | <.001 | 0.335 |
| Smoking, n (%) | 70 (13.28) | 62 (30.69) | <.001 | **0.377** |
| Drinking, n (%) | 63 (11.95) | 46 (22.77) | <.001 | 0.258 |
| Antibiotics, n (%) | 455 (86.34) | 190 (94.06) | 0.003 | 0.327 |
| Vasoactive drugs, n | 493 (93.55) | 94 (46.53) | <.001 | -0.943 |
| CKD, n (%) | 87 (16.51) | 9 (4.46) | <.001 | **-0.584** |
| SIRS, n (%) | 399 (75.71) | 17 (8.42) | <.001 | -2.424 |
| Sepsis, n (%) | 351 (66.60) | 31 (15.35) | <.001 | -1.422 |
| ACS, n (%) | 18 (3.42) | 22 (10.89) | <.001 | 0.240 |
| Shock, n (%) | 174 (33.02) | 32 (15.84) | <.001 | **-0.470** |
| Ventilation, n (%) | 270 (51.23) | 115 (56.93) | 0.168 | 0.115 |
| RRT, n (%) | 79 (14.99) | 72 (35.64) | <.001 | **0.431** |
| SOFA score | 2.00 ± 2.40 | 4.16 ± 3.90 | <.001 | 0.556 |
| CCI score | 3.89 ± 2.76 | 1.26 ± 1.31 | <.001 | -2.013 |
| WBC (x10^9^/L) | 13.67 ± 7.86 | 13.34 ± 6.92 | 0.600 | -0.048 |
| HCT (%) | 34.16 ± 7.31 | 36.66 ± 9.71 | 0.001 | 0.257 |
| PLT (x10^9^/L) | 215.67 ± 131.35 | 179.29 ± 91.03 | <.001 | -0.400 |
| Blood glucose(mg/dL) | 160.61 ± 118.75 | 214.59 ± 282.26 | 0.009 | 0.191 |
| Total bilirubin(mg/dL) | 2.45 ± 4.05 | 2.48 ± 3.01 | 0.913 | 0.011 |
| PT(s) | 16.91 ± 11.27 | 16.10 ± 5.30 | 0.193 | **-0.151** |
| APTT(s) | 35.82 ± 20.02 | 40.28 ± 13.42 | 0.004 | 0.333 |
| SCr (mg/dL) | 1.73 ± 1.85 | 1.80 ± 1.74 | 0.640 | 0.041 |
| BUN (mg/dL) | 30.33 ± 27.55 | 34.51 ± 25.29 | 0.061 | 0.165 |
| Calcium(mg/dL) | 7.90 ± 1.12 | 7.74 ± 1.76 | 0.225 | -0.092 |
| Sodium(mmol/L) | 138.35 ± 6.25 | 137.78 ± 6.41 | 0.277 | -0.088 |
| Potassium(mmol/L) | 4.17 ± 0.87 | 4.18 ± 0.71 | 0.954 | 0.005 |
| Chlorine(mmol/L) | 104.24 ± 7.87 | 101.25 ± 6.39 | <.001 | -0.468 |

Abbreviation: ACS, abdominal compartment syndrome; CKD, chronic kidney disease; RRT, renal replacement therapy; SIRS, systemic inflammatory response syndrome; SOFA, Sequential Organ Failure Assessment; CCI, Charlson Comorbidity Index; SMD, standard mean difference.


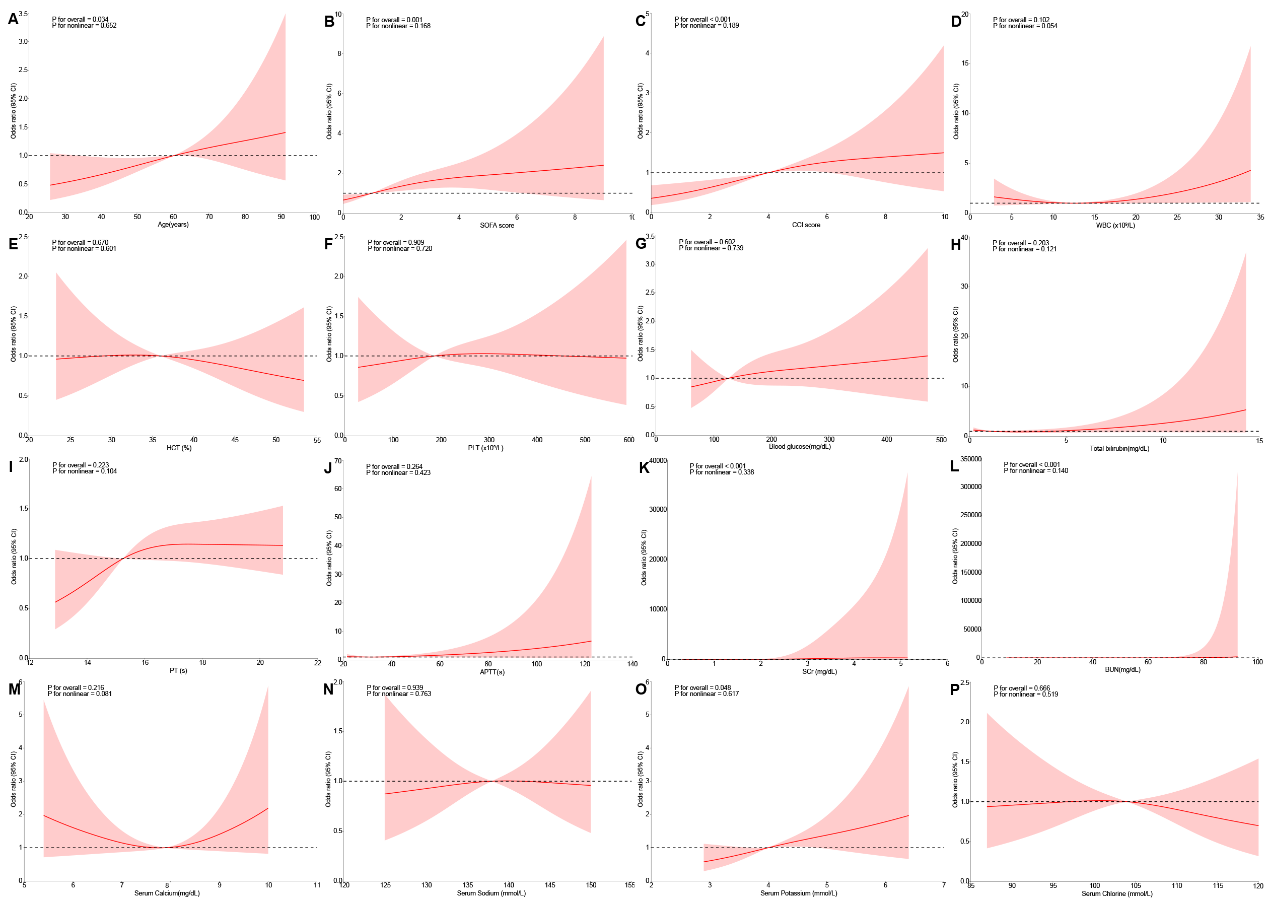


**Appendix 3.** Restricted cubic spline (RCS) curve for the continuous variables. The shaded ribbons represent 95% CIs. The horizontal dotted lines represent the hazard ratio of 1.0. (A) RCS for age; (B)RCS for SOFA score; (C)RCS for CCI score; (D)RCS for WBC; (E)RCS for HCT; (F)RCS for PLT; (G)RCS for blood glucose; (H)RCS for total bilirubin; (I)RCS for PT; (J)RCS for APTT; (K)RCS for SCr; (L)RCS for BUN; (M)RCS for serum calcium; (N)RCS for serum sodium; (O)RCS for serum potassium; (P)RCS for serum chlorine. CI, confidence interval; SOFA, Sequential Organ Failure Assessment; CCI, Charlson Comorbidity Index; WBC, white blood cell; HCT, hematocrit; PLT, platelet; PT, prothrombin time; APTT, activated partial thromboplastin time; SCr, serum creatinine; BUN, blood urea nitrogen.


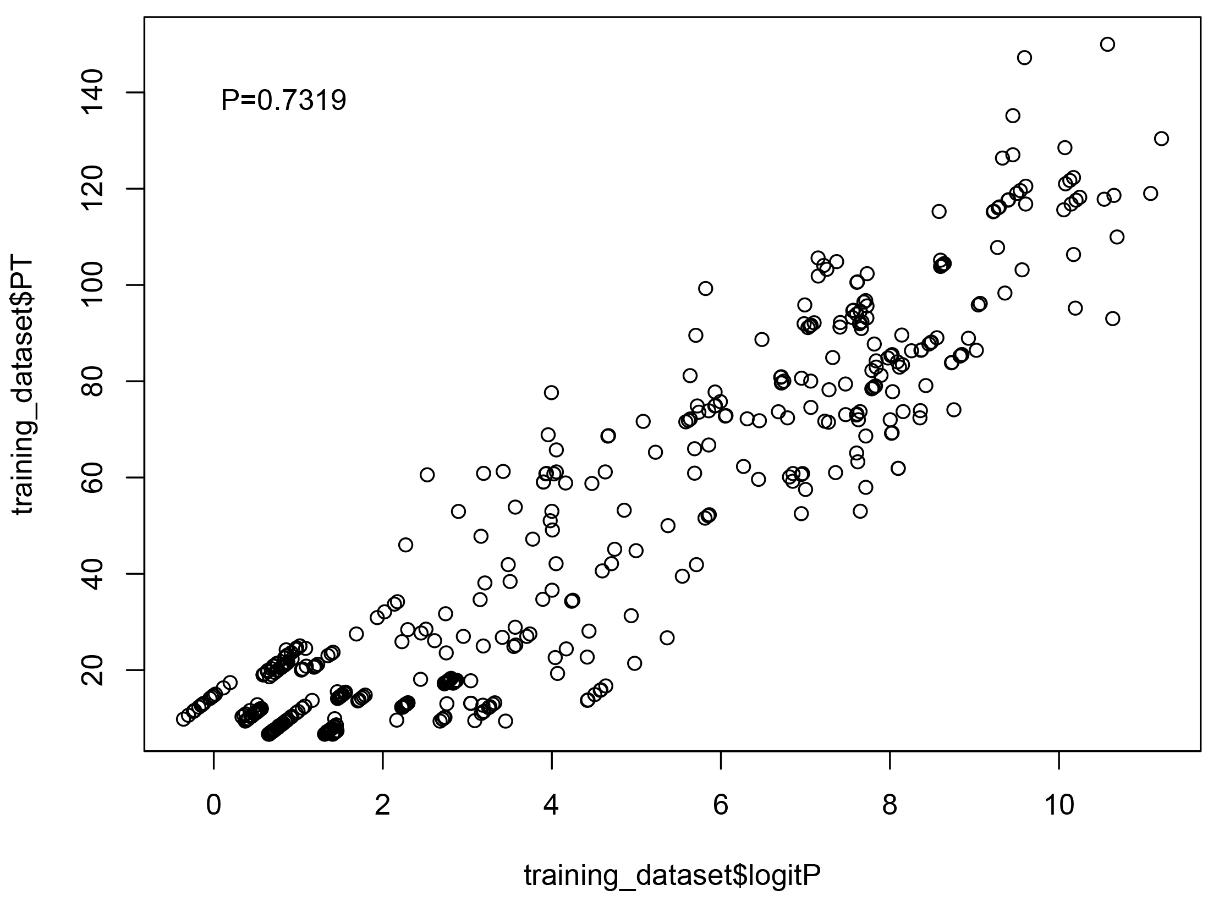


**Appendix 4.** Box-Tidwell test and scatter plot for continuous variable PT. PT, prothrombin time.


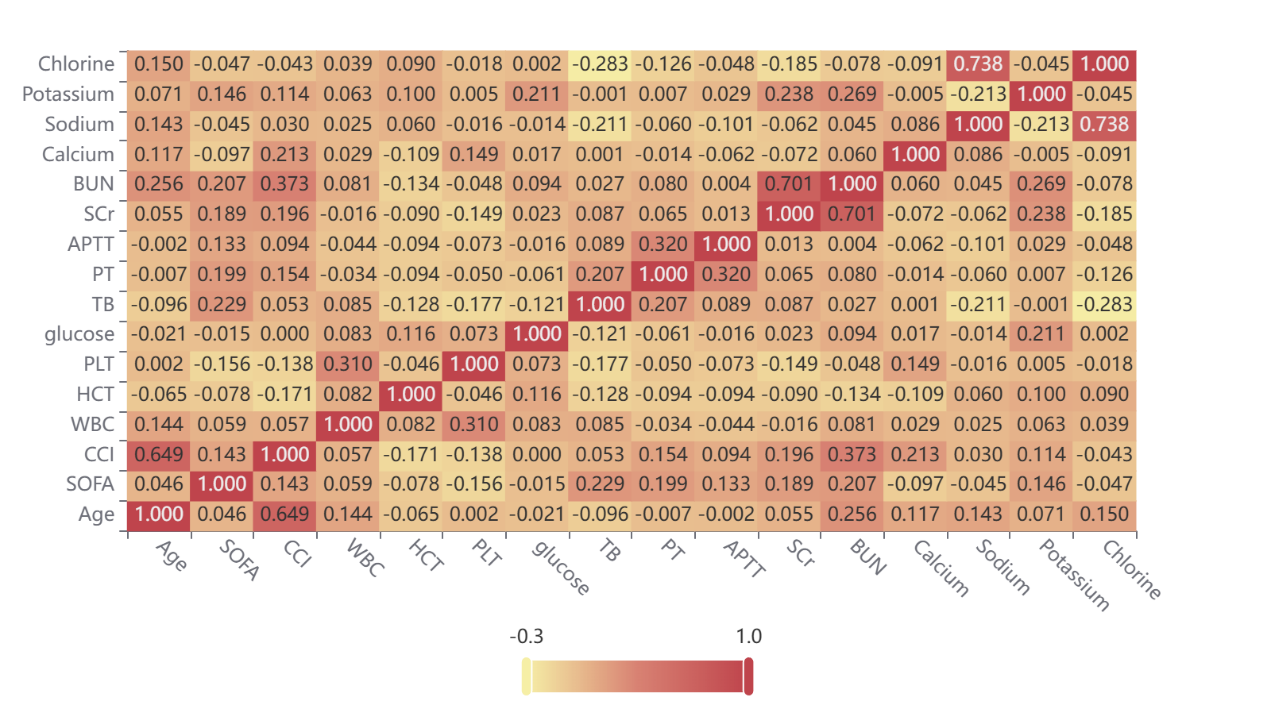


**Appendix 5.** Pearson correlation analysis heatmap for variables. SOFA, Sequential Organ Failure Assessment; CCI, Charlson Comorbidity Index; WBC, white blood cell; HCT, hematocrit; PLT, platelet; PT, prothrombin time; APTT, activated partial thromboplastin time; SCr, serum creatinine; BUN, blood urea nitrogen.

**Appendix 6.** The VIF values of variables

| Variables | VIF | Variables | VIF | Variables | VIF |
| --- | --- | --- | --- | --- | --- |
| Hyperlipidemia | 1.171 | Smoking | 1.037 | Antibiotics | 1.568 |
| CKD | 1.524 | SIRS | 1.102 | Sepsis | 1.821 |
| Shock | 1.317 | Ventilation | 1.374 | Age | 2.026 |
| SOFA | 1.257 | CCI | 2.304 | PT | 1.100 |
| SCr | 2.122 | BUN | 2.344 | Serum potassium | 1.124 |

Abbreviation: CKD, chronic kidney disease; SOFA, Sequential Organ Failure Assessment; CCI, Charlson Comorbidity Index; PT, prothrombin time; SCr, serum creatinine; BUN, blood urea nitrogen; VIF, variance inflation factor.


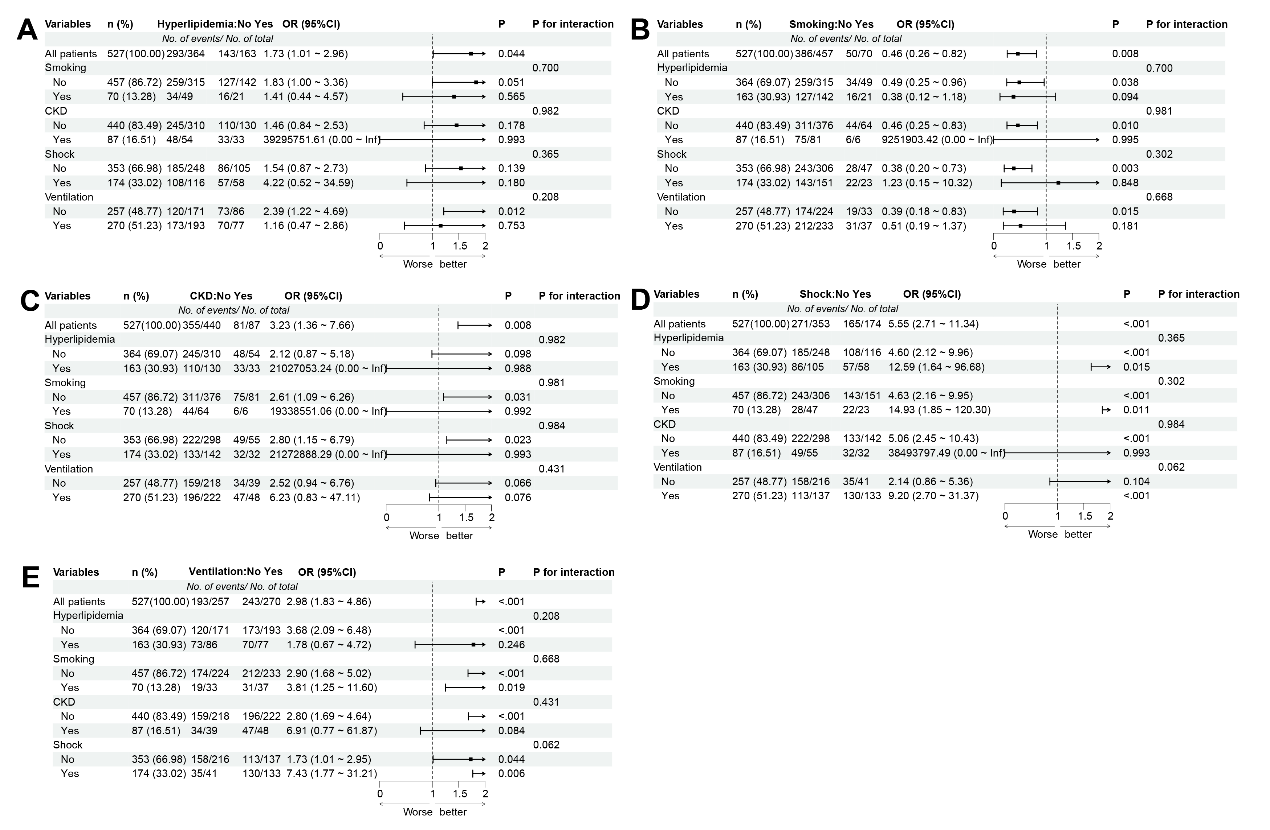


**Appendix 7.** Forest plots of subgroup analysis of the relationship between variables and AKI in patients with AP. (A) Forest plot for hyperlipidemia; (B) Forest plot for smoking; (C) Forest plot for CKD; (D) Forest plot for shock; (E) Forest plot for ventilation. Abbreviations: AP, acute pancreatitis; AKI, acute kidney injury; CKD, chronic kidney disease.
